# Supplementary material for: Sensory Features Introduced by Brewery Spent Grain with Impact on Consumers’ Motivations and Emotions for Fibre-Enriched Products
Source: Foods. 2021 Dec 24;11(1):36. doi: 10.3390/foods11010036 (PMC8750114; doi:10.3390/foods11010036)
Supplement: Supplementary file 1 [file foods-11-00036-s001.zip › Supplementary Questionnaire S1.pdf]

<u>Sensory evaluation of pasta</u>

You will receive two samples of pasta. Please answer the following questions and then evaluate the two samples.

Age ______

Gender

€ Female
€ Male
€ Preferred not to say

How often do you eat pasta?

€ 2-3 times a week
€ At least once a week
€ 1-2 times per month
€ Never

<u>Sample #1</u>

Please select one of the samples and write its three-digit code _______. First taste the sample and then answer the following questions.

<u>SECTION I</u>

1) How much do you like this sample?

	1	2	3	4	5	6	7	8	9	
I extremely dislike it	○	○	○	○	○	○	○	○	○	*I extremely like it*

2) From the following list, check all the attributes which best describe the sample. Choose all that apply.

€ Natural
€ Tasty
€ Soft
€ Fibrous
€ Uniform
€ Homogenous
€ Granulated

€ Coarse
€ Rubbery
€ Dark
€ Gritty
€ Lack of taste
€ Flexible
€ Sticky

€ Hard
€ Strange taste
€ Unpleasent aroma
€ Insipid
€ Unpleasent aftertase
€ Brittle

3) Would you buy this product?

	1	2	3	4	5	
I would definitely not buy it	○	○	○	○	○	*I would definitely buy it*

4) If your previous answer (Question 3) was 4 or 5, then why would you buy it? Choose as many answers as you see fit.

- € Because it is healthy
- € Because it is tasty
- € To avoid weight gain
- € Because it is high in calories
- € Because it is good for my family
- € Because I want to include fibre in my diet
- € Because my kids would like it

5) If your previous answer to Question 3 was 1, 2 or 3, then why wouldn't you buy it? Choose as many answers as you see fit.

- € Because it does not seem healthy
- € Because it is distasteful
- € Because it is not the one I always buy
- € Because I do not need fibre
- € To prevent weight gain
- € Because I think it is bad for my family
- € Because it seems expensive
- € Because I don't want to include fibre in my diet
- € Because my family would not like it

SECTION II

6) Select the intensity of the following emotions experienced after tasting the sample.

	Nothing	Slightly	Mildly	Very	Extremely
Active	€	€	€	€	€
Tame	€	€	€	€	€
Bored	€	€	€	€	€
Calm	€	€	€	€	€
Disgusted	€	€	€	€	€
Mild	€	€	€	€	€
Enthusiastic	€	€	€	€	€
Worried	€	€	€	€	€
Free	€	€	€	€	€
Good	€	€	€	€	€
Good natured	€	€	€	€	€
Guilty	€	€	€	€	€
Adventurous	€	€	€	€	€
Happy	€	€	€	€	€
Interested	€	€	€	€	€
Secure	€	€	€	€	€

Joyful	€	€	€	€	€
Loving	€	€	€	€	€
Wild	€	€	€	€	€
Nostalgic	€	€	€	€	€
Pleasant	€	€	€	€	€
Satisfied	€	€	€	€	€
Understanding	€	€	€	€	€
Warm	€	€	€	€	€
Aggressive	€	€	€	€	€

Sample #2

Please select the other sample and write its three-digit code _______. First taste the sample and then answer the following questions.

SECTION I

1) How much do you like this sample?

	1	2	3	4	5	6	7	8	9	
I extremely dislike it	○	○	○	○	○	○	○	○	○	*I extremely like it*

2) From the following list, check all the attributes which best describe the sample. Choose all that apply.

€ Natural
€ Tasty
€ Soft
€ Fibrous
€ Uniform
€ Homogenous
€ Granulated
€ Coarse
€ Rubbery
€ Dark
€ Gritty
€ Lack of taste
€ Flexible
€ Sticky
€ Hard
€ Strange taste
€ Unpleasent aroma
€ Insipid
€ Unpleasent aftertase
€ Brittle

3) Would you buy this product?

	1	2	3	4	5	
I would definitely not buy it	○	○	○	○	○	*I would definitely buy it*

4) If your previous answer (Question 3) was 4 or 5, then why would you buy it? Choose as many answers as you see fit.

€ Because it is healthy
€ Because it is tasty
€ To avoid weight gain

- € Because it is high in calories
- € Because it is good for my family
- € Because I want to include fibre in my diet
- € Because my kids would like it

5) If your previous answer to Question 3 was 1, 2 or 3, then why wouldn't you buy it? Choose as many answers as you see fit.

- € Because it does not seem healthy
- € Because it is distasteful
- € Because it is not the one I always buy
- € Because I do not need fibre
- € To prevent weight gain
- € Because I think it is bad for my family
- € Because it seems expensive
- € Because I don't want to include fibre in my diet
- € Because my family would not like it

<u>SECTION II</u>

6) Select the intensity of the following emotions experienced after tasting the sample.

	**Nothing**	**Slightly**	**Mildly**	**Very**	**Extremely**
Bored	€	€	€	€	€
Active	€	€	€	€	€
Warm	€	€	€	€	€
Pleasant	€	€	€	€	€
Aggressive	€	€	€	€	€
Joyful	€	€	€	€	€
Loving	€	€	€	€	€
Disgusted	€	€	€	€	€
Adventurous	€	€	€	€	€
Good	€	€	€	€	€
Good natured	€	€	€	€	€
Calm	€	€	€	€	€
Understanding	€	€	€	€	€
Guilty	€	€	€	€	€
Enthusiastic	€	€	€	€	€
Happy	€	€	€	€	€
Tame	€	€	€	€	€
Interested	€	€	€	€	€
Mild	€	€	€	€	€
Free	€	€	€	€	€
Nostalgic	€	€	€	€	€
Worried	€	€	€	€	€

Wild	€	€	€	€	€
Satisfied	€	€	€	€	€
Secure	€	€	€	€	€

Thank you!

Sensory evaluation of chocolate milk

You will receive two samples of chocolate milk. Please answer the following questions and then evaluate the two samples.

Age ______

Gender

- € Female
- € Male
- € Preferred not to say

How often do you eat pasta?

- € Every day
- € At least once a week
- € At least once a month
- € Never

Sample #1

Please select one of the samples and write its three-digit code _______. First taste the sample and then answer the following questions.

SECTION I

1) How much do you like this sample?

	1	2	3	4	5	6	7	8	9	
I extremely dislike it	○	○	○	○	○	○	○	○	○	*I extremely like it*

2) How did you feel about the consistency of this product?

Much too thin	2	3	*Just about right*	5	6	*Much too thick*
○	○	○	○	○	○	○

3) How did you feel about the flavour of this product?

Much too little		3	*Just about right*	5	6	*Much too much*
○	○	○	○	○	○	○

4) How did you feel about the sweetness of this product?

Not at all Sweet enough	2	3	*Just about right*	5	6	*Much too Sweet*
○	○	○	○	○	○	○

5) From the following list, check all the attributes which best describe the sample. Choose all that apply.

- € Smooth
- € Strong chocolate flavour
- € Natural
- € Dark
- € Gritty
- € Bitter
- € Strong chocolate aroma
- € Artificial
- € Coarse
- € Creamy
- € unpleasant Aftertaste
- € Strange taste
- € Healthy
- € Milk flavour
- € Unpleasant Aroma
- € Greasy
- € Lacking taste
- € Pale
- € Acidic
- € Bland

6) Would you buy this product?

I would definitely not buy it	1	2	3	4	5	*I would definitely buy it*
	○	○	○	○	○	

7) If your previous answer (Question 6) was 4 or 5, then why would you buy it? Choose as many answers as you see fit.

- € Because it is healthy
- € Because it is tasty
- € To avoid weight gain
- € Because it is high in calories
- € Because it is good for my family
- € Because I want to include fibre in my diet
- € Because my kids would like it

8) If your previous answer to Question 6 was 1, 2 or 3, then why wouldn't you buy it? Choose as many answers as you see fit.

- € Because it does not seem healthy
- € Because it is distasteful
- € Because it is not the one I always buy
- € Because I do not need fibre
- € To prevent weight gain
- € Because I think it is bad for my family
- € Because it seems expensive

- € Because I don't want to include fibre in my diet
- € Because my family would not like it

SECTION II

9) Select the intensity of the following emotions experienced after tasting the sample.

	Nothing	Slightly	Mildly	Very	Extremely
Worried	€	€	€	€	€
Good	€	€	€	€	€
Interested	€	€	€	€	€
Happy	€	€	€	€	€
Active	€	€	€	€	€
Guilty	€	€	€	€	€
Good natured	€	€	€	€	€
Free	€	€	€	€	€
Satisfied	€	€	€	€	€
Pleasant	€	€	€	€	€
Warm	€	€	€	€	€
Nostalgic	€	€	€	€	€
Wild	€	€	€	€	€
Mild	€	€	€	€	€
Loving	€	€	€	€	€
Joyful	€	€	€	€	€
Bored	€	€	€	€	€
Aggressive	€	€	€	€	€
Enthusiastic	€	€	€	€	€
Disgusted	€	€	€	€	€
Understanding	€	€	€	€	€
Tame	€	€	€	€	€
Secure	€	€	€	€	€
Calm	€	€	€	€	€
Adventurous	€	€	€	€	€

Sample #2

Please select the other sample and write its three-digit code _______. First taste the sample and then answer the following questions.

SECTION I

1) How much do you like this sample?

	1	2	3	4	5	6	7	8	9	
I extremely dislike it	○	○	○	○	○	○	○	○	○	*I extremely like it*

2) How did you feel about the consistency of this product?

Much too thin	2	3	*Just about right*	5	6	*Much too thick*
○	○	○	○	○	○	○

3) How did you feel about the flavour of this product?

Much too little		3	*Just about right*	5	6	*Much too much*
○	○	○	○	○	○	○

4) How did you feel about the sweetness of this product?

Not at all Sweet enough	2	3	*Just about right*	5	6	*Much too Sweet*
○	○	○	○	○	○	○

5) From the following list, check all the attributes which best describe the sample. Choose all that apply.

€ Lacking taste
€ Bitter
€ Coarse
€ Smooth
€ Strange taste
€ Milk flavour
€ Natural
€ Pale
€ Unpleasant Aroma
€ Creamy
€ Strong chocolate aroma
€ Strong chocolate flavour
€ Bland
€ Acidic
€ Artificial
€ Dark
€ Greasy
€ Gritty
€ Unpleasant Aftertaste
€ Healthy

6) Would you buy this product?

	1	2	3	4	5	
I would definitely not buy it	○	○	○	○	○	*I would definitely buy it*

7) If your previous answer (Question 6) was 4 or 5, then why would you buy it? Choose as many answers as you see fit.

€ Because it is healthy

- € Because it is tasty
- € To avoid weight gain
- € Because it is high in calories
- € Because it is good for my family
- € Because I want to include fibre in my diet
- € Because my kids would like it

8) If your previous answer to Question 6 was 1, 2 or 3, then why wouldn't you buy it? Choose as many answers as you see fit.

- € Because it does not seem healthy
- € Because it is distasteful
- € Because it is not the one I always buy
- € Because I do not need fibre
- € To prevent weight gain
- € Because I think it is bad for my family
- € Because it seems expensive
- € Because I don't want to include fibre in my diet
- € Because my family would not like it

SECTION II

9) Select the intensity of the following emotions experienced after tasting the sample.

	**Nothing**	**Slightly**	**Mildly**	**Very**	**Extremely**
Joyful	€	€	€	€	€
Free	€	€	€	€	€
Satisfied	€	€	€	€	€
Pleasant	€	€	€	€	€
Guilty	€	€	€	€	€
Good natured	€	€	€	€	€
Worried	€	€	€	€	€
Wild	€	€	€	€	€
Mild	€	€	€	€	€
Loving	€	€	€	€	€
Enthusiastic	€	€	€	€	€
Disgusted	€	€	€	€	€
Understanding	€	€	€	€	€
Warm	€	€	€	€	€
Nostalgic	€	€	€	€	€
Good	€	€	€	€	€
Interested	€	€	€	€	€
Bored	€	€	€	€	€
Aggressive	€	€	€	€	€
Secure	€	€	€	€	€
Calm	€	€	€	€	€

Adventurous	€	€	€	€	€
Happy	€	€	€	€	€
Active	€	€	€	€	€
Tame	€	€	€	€	€

Thank you!

<u>Sensory evaluation of bread</u>

You will receive two samples of bread. Please answer the following questions and then evaluate the two samples.

Age ______

Gender

- € Female
- € Male
- € Preferred not to say

How often do you eat bread?

- € 3-4 times a week
- € At least once a week
- € At least once a month
- € Never

<u>Sample #1</u>

Please select one of the samples and write its three-digit code _______. First taste the sample and then answer the following questions.

<u>SECTION I</u>

1) How much do you like this sample?

	1	2	3	4	5	6	7	8	9	
I extremely dislike it	○	○	○	○	○	○	○	○	○	*I extremely like it*

2) How did you feel about the <u>saltiness</u> of this product?

Much too little	2	3	*Just about right*	5	6	*Much too much*
○	○	○	○	○	○	○

3) From the following list, check all the attributes which best describe the sample. Choose all that apply.

- € Fibrous
- € Soft
- € Natural
- € Compact
- € Fluffy
- € Dry
- € Moist
- € Strange taste
- € Stodgy
- € Bitter
- € Airy
- € Hard to swallow
- € Unpleasant aroma
- € Unpleasant aftertaste
- € Salty
- € Hard
- € Hard to chew
- € Bland
- € Sweet
- € Crispy
- € Acidic

4) Would you buy this product?

	1	2	3	4	5	
I would definitely not buy it	○	○	○	○	○	*I would definitely buy it*

5) If your previous answer (Question 4) was 4 or 5, then why would you buy it? Choose as many answers as you see fit.

€ Because it is healthy
€ Because it is tasty
€ To avoid weight gain
€ Because it is high in calories
€ Because it is good for my family
€ Because I want to include fibre in my diet
€ Because my kids would like it

6) If your previous answer to Question 4 was 1, 2 or 3, then why wouldn't you buy it? Choose as many answers as you see fit.

€ Because it does not seem healthy
€ Because it is distasteful
€ Because it is not the one I always buy
€ Because I do not need fibre
€ To prevent weight gain
€ Because I think it is bad for my family
€ Because it seems expensive
€ Because I don't want to include fibre in my diet
€ Because my family would not like it

<u>SECTION II</u>

7) Select the intensity of the following emotions experienced after tasting the sample.

	**Nothing**	**Slightly**	**Mildly**	**Very**	**Extremely**
Wild	€	€	€	€	€
Mild	€	€	€	€	€
Pleasant	€	€	€	€	€
Guilty	€	€	€	€	€
Good natured	€	€	€	€	€
Worried	€	€	€	€	€
Loving	€	€	€	€	€
Nostalgic	€	€	€	€	€
Good	€	€	€	€	€
Aggressive	€	€	€	€	€
Secure	€	€	€	€	€
Calm	€	€	€	€	€

Adventurous	€	€	€	€	€
Happy	€	€	€	€	€
Active	€	€	€	€	€
Tame	€	€	€	€	€
Interested	€	€	€	€	€
Bored	€	€	€	€	€
Joyful	€	€	€	€	€
Enthusiastic	€	€	€	€	€
Disgusted	€	€	€	€	€
Understanding	€	€	€	€	€
Warm	€	€	€	€	€
Free	€	€	€	€	€
Satisfied	€	€	€	€	€

Sample #2

Please select the other sample and write its three-digit code _______. First taste the sample and then answer the following questions.

SECTION I

1) How much do you like this sample?

	1	2	3	4	5	6	7	8	9	
I extremely dislike it	○	○	○	○	○	○	○	○	○	*I extremely like it*

2) How did you feel about the saltiness of this product?

Much too little	2	3	*Just about right*	5	6	*Much too much*
○	○	○	○	○	○	○

3) From the following list, check all the attributes which best describe the sample. Choose all that apply.

€ Dry	€ Strange taste	€ Soft
€ Airy	€ Bland	€ Natural
€ Hard to swallow	€ Sweet	€ Compact
€ Unpleasant aftertaste	€ Crispy	€ Fluffy
€ Salty	€ Acidic	€ Moist
€ Hard	€ Unpleasant aroma	€ Stodgy
€ Hard to chew	€ Fibrous	€ Bitter

4) Would you buy this product?

	1	2	3	4	5	
I would definitely not buy it	○	○	○	○	○	*I would definitely buy it*

5) If your previous answer (Question 4) was 4 or 5, then why would you buy it? Choose as many answers as you see fit.

€ Because it is healthy
€ Because it is tasty
€ To avoid weight gain
€ Because it is high in calories
€ Because it is good for my family
€ Because I want to include fibre in my diet
€ Because my kids would like it

6) If your previous answer to Question 4 was 1, 2 or 3, then why wouldn't you buy it? Choose as many answers as you see fit.

€ Because it does not seem healthy
€ Because it is distasteful
€ Because it is not the one I always buy
€ Because I do not need fibre
€ To prevent weight gain
€ Because I think it is bad for my family
€ Because it seems expensive
€ Because I don't want to include fibre in my diet
€ Because my family would not like it

SECTION II

7) Select the intensity of the following emotions experienced after tasting the sample.

	**Nothing**	**Slightly**	**Mildly**	**Very**	**Extremely**
Satisfied	€	€	€	€	€
Pleasant	€	€	€	€	€
Good natured	€	€	€	€	€
Disgusted	€	€	€	€	€
Understanding	€	€	€	€	€
Warm	€	€	€	€	€
Free	€	€	€	€	€
Worried	€	€	€	€	€
Loving	€	€	€	€	€
Nostalgic	€	€	€	€	€
Good	€	€	€	€	€
Wild	€	€	€	€	€

Active	€	€	€	€	€
Aggressive	€	€	€	€	€
Secure	€	€	€	€	€
Calm	€	€	€	€	€
Adventurous	€	€	€	€	€
Happy	€	€	€	€	€
Guilty	€	€	€	€	€
Tame	€	€	€	€	€
Interested	€	€	€	€	€
Bored	€	€	€	€	€
Mild	€	€	€	€	€
Joyful	€	€	€	€	€
Enthusiastic	€	€	€	€	€

Thank you!
