# Supplementary material for: Sensory Features Introduced by Brewery Spent Grain with Impact on Consumers’ Motivations and Emotions for Fibre-Enriched Products
Source: Foods. 2021 Dec 24;11(1):36. doi: 10.3390/foods11010036 (PMC8750114; doi:10.3390/foods11010036)
Supplement: Supplementary file 1 [file foods-11-00036-s001.zip › Supplementary Table S1.pdf]

Supplementary Table S1

Nutritional facts of fibre-enriched and regular bread, pasta and chocolate milk expressed in 100 g of product.

	Fibre-enriched bread	Regular bread	Fibre-enriched pasta	Regular pasta	Fibre-enriched chocolate milk	Regular chocolate milk
Energy (kcal)	284	296	273	276	76	75
Proteins (g)	9.6	8.4	11.3	10.9	3.1	3.0
Lipids (g)	7.5	7.0	2.5	2.3	1.7	1.7
Dietary fibre (g)*	5.8	1.8	3.4	1.5	1.3	1.1
Carbohydrates[1] (g)	44.4	49.8	51.4	53.0	12.0	11.9

[1] - Carbohydrate content was obtained by difference.

*Source of fibre claim in EU Regulation (EC) No 1924/2006, could be used when product has 3 g/100g of dietary fibre or 1.5 g of dietary fibre/100 kcal.
